# A dataset of human body tracking of walking actions captured using two Azure Kinect sensors

**DOI:** 10.1016/j.dib.2023.109334

**Published:** 2023-06-22

**Authors:** Charli Posner, Adrián Sánchez-Mompó, Ioannis Mavromatis, Mustafa Al-Ani

**Affiliations:** Bristol Research and Innovation Laboratory, Toshiba Europe Ltd., 32 Queen Square, Bristol, BS1 4ND, United Kingdom

**Keywords:** Computer vision, Machine learning, Body tracking, Human motion prediction, Gait analysis, Walking, Dataset, Azure Kinect

## Abstract

A dataset of body tracking information is presented. The dataset consists of 315 captured walking sequences. Each sequence is simultaneously captured by two Azure Kinect devices. The two captures are interleaved to effectively double the frame rate. Fifteen participants partook in this experiment. Each experiment consists of seven walking actions, and having three predefined trajectories per experiment. That results in 21 sequences per participant. The data were collected using the Azure Kinect Sensor SDK. They were later processed using the official tools and libraries provided by Microsoft. For each sequence and trajectory, the positions and orientations of thirty-two tracked joints were obtained and saved.

The dataset is structured as follows. The experiments from each subject are saved in a single directory. Each directory contains multiple JSON files of timestamped body tracking information to enable the fusion of the two device streams. A calibration file is also provided, enabling the mapping of the coordinates between the two Azure Kinect devices capturing the data (mapping the coordinates of the device known as the Subordinate device to the Master device coordinate system). This data can be used to train neural networks for human motion prediction tasks or test pre-existing algorithms on Azure Kinect data. This dataset could also aid in gait recognition and analysis, as well as in performing action recognition and other surveillance activities. The dataset can be found at https://zenodo.org/record/7997856.

**Specifications table**

**Table:** 

Subject	Computer Science
Specific subject area	Human Motion Prediction and Computer Vision
Type of data	Azure Kinect [1] body tracking information is provided as JSON [2] files. Each file contains skeletal data for thirty-two different tracked joints of the human body. Each joint is tracked for all video frames where a human body is visible. Each JSON file is paired with a TXT file containing system timestamps for each frame for the RGB, Depth and IR sensors in nanoseconds - the time recorded when the image processing was concluded. A series of accompanying TXT files provide the timing of the slave and master captures for each sequence and the sequence length in frames of body tracking information for each video. A separate unique TXT file contains calibration information common for the whole dataset.
How the data were acquired	Two Azure Kinects were set up in a laboratory, and three walking trajectories were mapped within their FoV. A stereo calibration was initially performed to find a coordinate mapping between the sensors. The participants later performed seven walking actions each for all different trajectories. Videos were captured using a modified version of Microsoft's *k*4*arecorder*[3] library, which allows us to obtain a system timestamp on arrival from the device. This enables the interleaving of sensor captures. Video feeds were later processed using a modified version of Microsoft's *offline processor*[4] to obtain JSON files of body tracking coordinates with respect to the focal point of the colour camera.
Data format	Raw and Pre-processed
Description of data collection	The data were collected in a laboratory set-up, in which participants performed each of the walking actions three times, once along each of three trajectories while being across the Kinect's FoV. Two videos and corresponding system timestamps were captured for each sequence. The devices’ capture times were synchronised externally using an Arduino Uno, such that the frame captures were interleaved.
Data source location	• Institution: Toshiba Bristol Research and Innovation Laboratory • City/Town/Region: Bristol • Country: United Kingdom
Data accessibility	Repository name: Zenodo Data identification number: DOI:10.5281/zenodo.7997856 Direct URL to data: https://zenodo.org/record/7997856

## Value of the Data

• The dataset consists of 630 JSON files (2 files - one per Kinect sensor - for each of the 315 recorded sequences). There are also 630 corresponding TXT files containing body tracking and system timestamp information, respectively. Body tracking sequence lengths are also provided and range from 60-160 frames for each video. By capturing a large number of sequences, using numerous subjects with different walking styles and speeds, the dataset is diverse in nature and applicable to many walking prediction use-cases.
• This is the only Microsoft Kinect dataset with a doubled system frame rate of 60 fps, achieved by an external synchronisation device (Arduino Uno) interleaving captures. With included system timestamps and calibration information, the streams can be combined effectively, improving the robustness of Azure Kinect body tracking and allowing for interpolation to a higher frame rate.
• Our dataset is unique among other Kinect datasets, including action classes designed for real-world walking behaviour. The seven recorded action classes provide ample data for action recognition tasks and allow for the creation of motion forecasting algorithms. The diverse participants, with differences in age, gender, and cultural background, also ensure unbiased algorithms can be trained using this dataset.
• Finally, increasing the window of prediction of the motion forecasting algorithms, this dataset can be utilised to train machine learning algorithms for human motion prediction over longer periods of time, as done in [5] with video sequences.

## 1 Objective

We have a need for a robust, inexpensive, and high refresh rate solution for body tracking in scenarios like the ones described in the action classes in this dataset to aid with the focusing of a mmWave radar imaging system. As we haven't found any solution that accomplishes all of our objectives to the level required whilst keeping the cost to an acceptable level, we decided to produce this setup to allow us to increase the frame rate and the robustness of the Azure Kinect body tracking system. Furthermore, we produced this dataset to train a Long Short-Term Memory (LSTM) neural network to predict future movements of the human joints on different scenarios to increase the robustness of the system.

## 2 Data Description

For data collection, three trajectories were marked on the floor across the Kinect FoV, labelled A, B and C. The first is perpendicular to the sensor, the second moving away from the sensor on an angle, and the third coming closer, as shown in Fig. 9(a). All subjects started from the corresponding floor marker position on the left of the FoV and walked across the trajectory during capture. All sequences were recorded for 10 seconds, equating to 300 frames captured per video. Each subject performed all 7 walking actions once per trajectory, resulting in 21 walking sequences and 42 video streams per person.

**Fig. 1:**
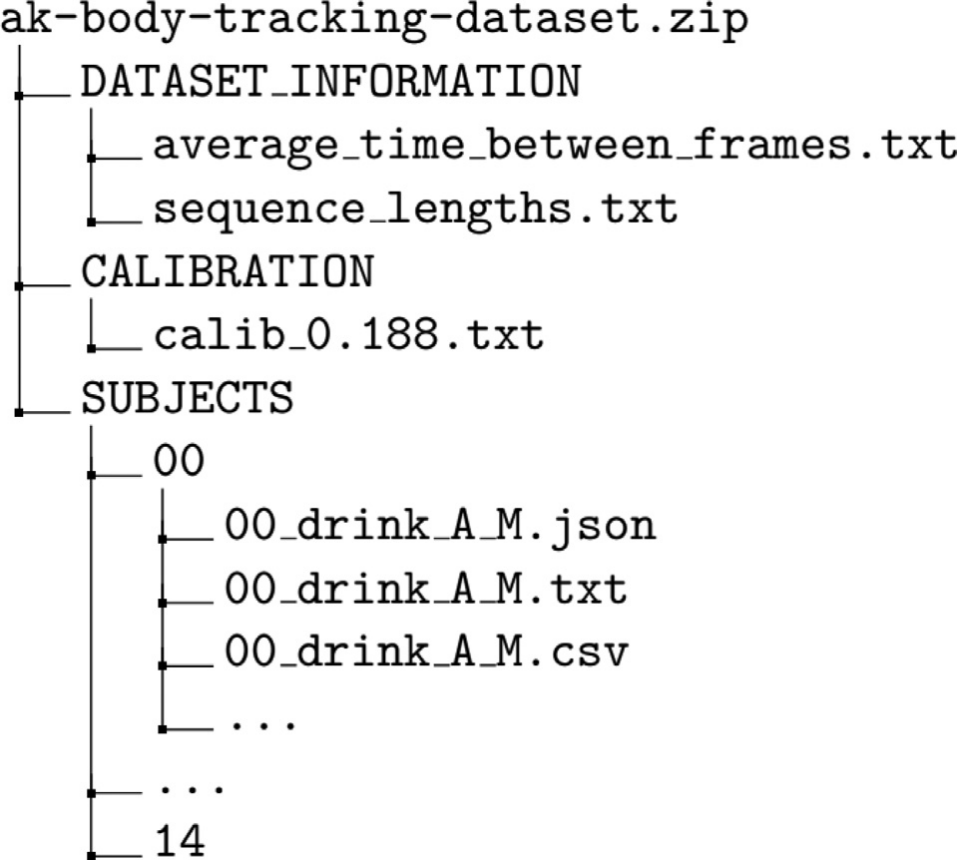
A directory tree depicting the structure of the dataset Zip file.

The dataset is organised according to the directory tree in Fig. 1. The dataset is uploaded as a single Zip file, unfolding into three directories, i.e., SUBJECTS, CALIBRATION, and DATASET_INFORMATION. The SUBJECTS directory contains the body tracking data. The CALIBRATION folder holds the coordinate transformation matrices mapping between the sensors. Finally, the DATASET_INFORMATION groups all the dataset-related information, such as the average time between frames of the different sequences in nanoseconds and the length of each sequence in frames. Within the SUBJECTS directory, one can find 15 sub-directories, one per experiment participant. Each sub-directory (named XX and ranging from 00 to 14) contains 84 files - 42 JSON and 42 TXT files. The numbering scheme described is used for anonymising the data from each participant. The JSON files contain the walking sequence (one for each sensor), and the TXT files the corresponding system timestamp. Further, a calibrated sequence for the Master and Subordinate devices to translate them to a common coordinate space can be found under the same directory in the CSV files with the same naming.

**Fig. 2:**
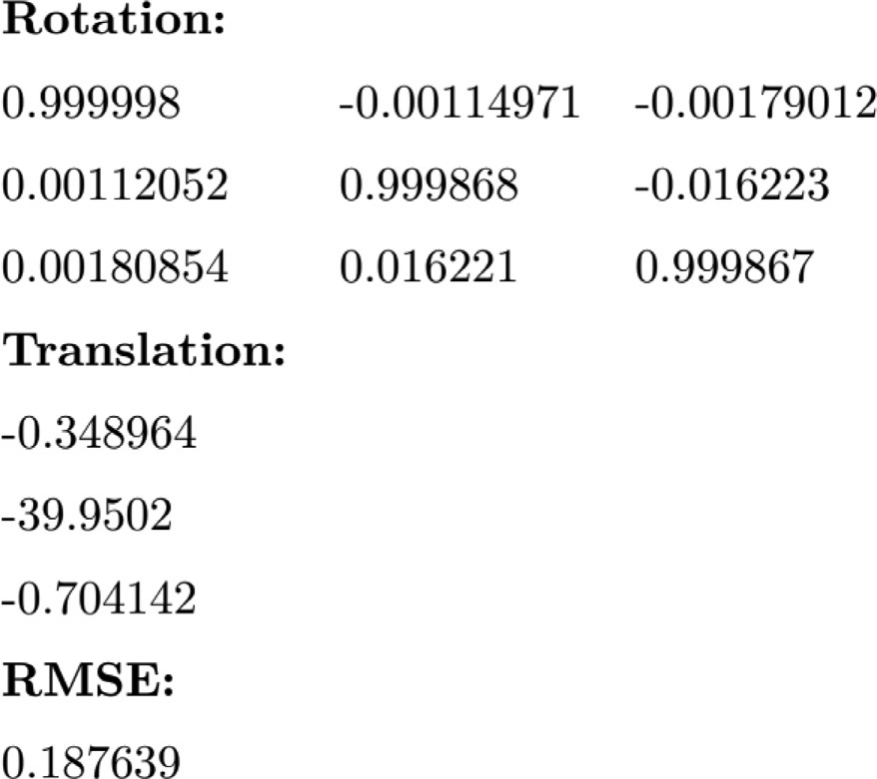
Contents of an example Calibration file: calib 0.188.txt.

Participants’ data is labelled according to the key: {SUBJECT}_{ACTION}_{TRAJECTORY}_{SENSOR}. For example, the file “00_drink_A_M.json” contains body tracking information of subject 00, performing the drink action and walking along trajectory A, as captured by the Master sensor. This key is used for both JSON and TXT files. The dataset contains one calibration file, containing a 3 *×* 3 rotation matrix, a 3 *×* 1 translation vector, and the Root Mean Squared Error (RMSE) of re-projection from Subordinate (S) to Master (M) coordinate systems. The calibration file is labelled according to the key: calib_{RMSE} - for example, “calib_0.188.txt” contains calibration rotation and translation matrices mapping S to M, such that the RMSE of re-projection is 0.188 pixels. System timestamp TXT files contain each of the colour, depth and infrared image timestamps for each frame, with the depth and infrared times being equal for all frames, as they use the same sensor. The rotation matrix and the translation vector format used in the calibration file can be seen in Fig. 2.

**Fig. 3:**
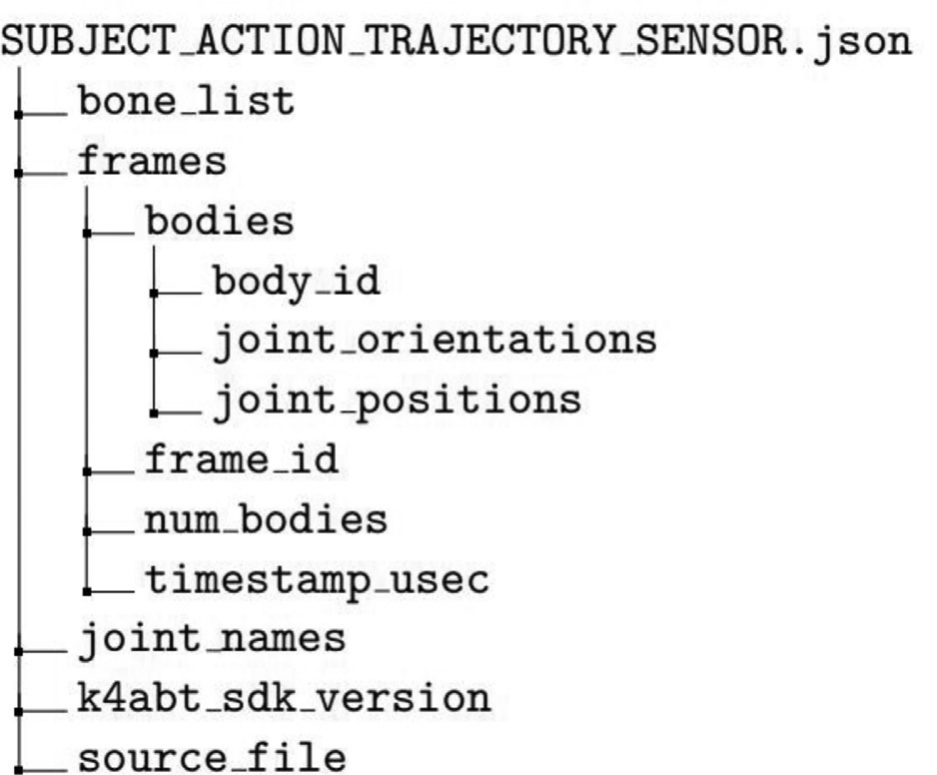
A directory tree depicting the structure of each JSON file of body tracking information.

The body tracking JSON files are structured as shown in Fig. 3. If a body is not detected in a given frame, the *bodies* directory will contain an empty list, and *num_bodies* will be 0. For frames in which a body is detected, the *bodies* directory will contain the orientations and positions of all 32 joints in the order given in the *joint_names* directory. The definitions of each item in the JSON structure are shown in Table 1.

**Table 1:** Definition the field names in the dataset JSON files.

Field Name	Definition
bone_list	A list of pairs of joints connected in the Microsoft Body Skeletal Tracking
frames	A list containing frame information for all captured frames
bodies	A list containing information for all detected bodies in a frame. No bodies = empty list
frame_id	The frame ID of the current frame
num_bodies	The number of bodies detected in the given frame
timestamp_usec	The device timestamp recorded when the frame is captured^2^
body_id	The ID of each body detected within the frame
joint_orientations	A list of quaternions representing joint orientations - one for each joint
joint_positions	A list of coordinates (in mm) representing joint positions - one for each joint
joint_names	A list of all the joint names in the Body Tracking skeleton, in order
k4abt_sdk_version	The version of the Body Tracking SDK used when processing videos (we used v1.1.1)
source_file	The name of the Matroska video file used to generate the JSON body tracking file

2 Device time is local to each sensor, rather than universal like system time. This is why the inclusion of system timestamps is necessary for the comparison and combination of device streams.

## 3 Experimental Design, Materials and Methods

To capture the dataset, two Azure Kinects were synchronised, and their captures were interleaved captures. The following steps were followed for that:

• Calibrate sensors to generate coordinate transformation.
• Configure external synchronisation system.
• Set up laboratory for data collection.
• Capture videos of walking sequences.
• Post-process videos to obtain body tracking information.

**Fig. 4:**
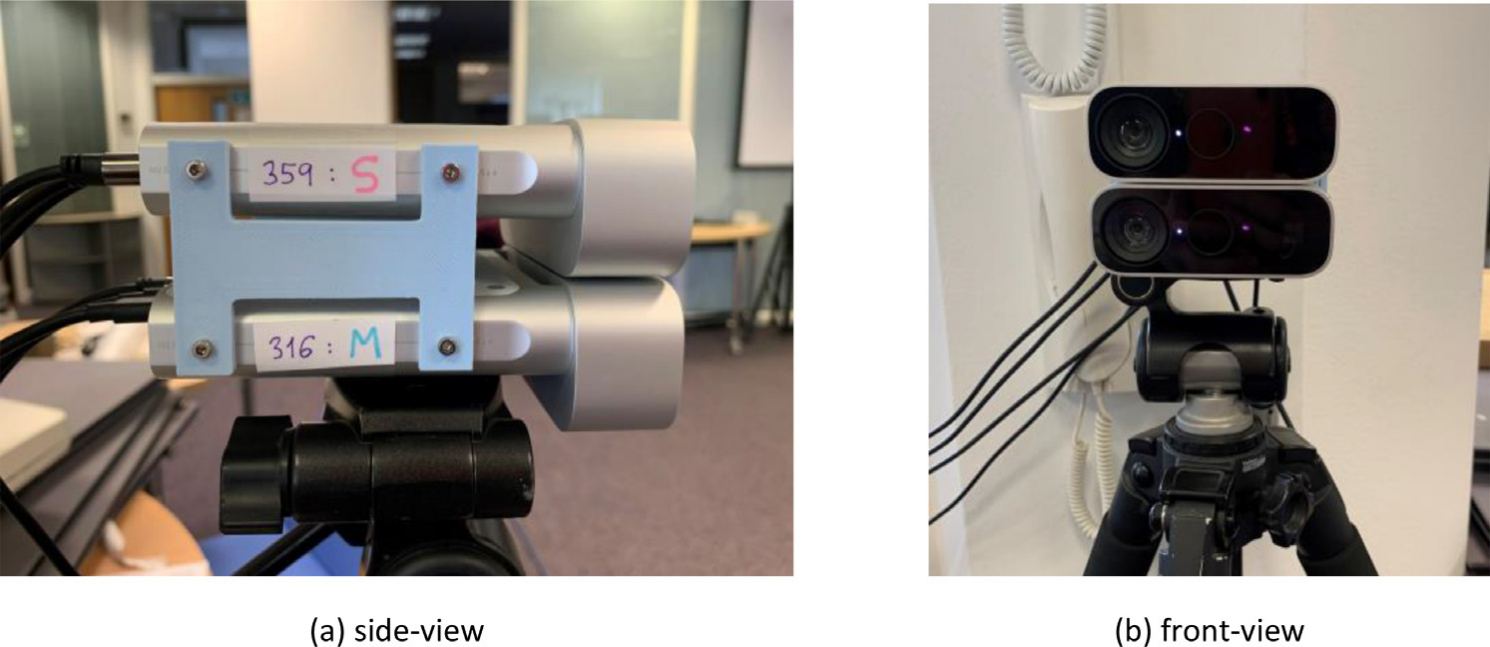
The Azure Kinects were attached together using 3D-printed brackets, separated by a 1mm gap as shown in (a), and mounted onto a tripod, as shown in (b). They were also labelled according to their mode during calibration (Master/Subordinate) and their serial numbers.

Table 2 shows the configurations used for both Azure Kinect devices throughout the investigation. The experiment was conducted in a laboratory space in Toshiba's Bristol Research and Innovation Laboratory (BRIL) offices. The sensors were mounted onto a tripod, one atop the other, and fixed together using 3D-printed brackets designed according to hardware measurements (Fig. 4). The configuration was such that a 1*mm* gap separated the devices, positioning the RGB and IR/depth sensors 4cm apart. The distance to the ground from the lowest device was 1*.*2*m*, and the Azure Kinects were completely parallel to the floor. In addition, although both devices acted as Subordinates during data collection, with the Arduino providing the Master signal, they were labelled as Master and Subordinate according to how they were calibrated. This made it clear which of the sensors’ coordinates needed to be transformed with the calibration matrices in post-processing.

**Table 2:** Sensor configuration used to capture dataset.

Depth mode	Narrow field-of-view (NFOV) Unbinned
RGB aspect ratio	4:3
RGB resolution	1536p
Frame rate	30fps
Exposure	0 (capped at frame period of sensor)
Firmware version	1.6.11

**Fig. 5:**
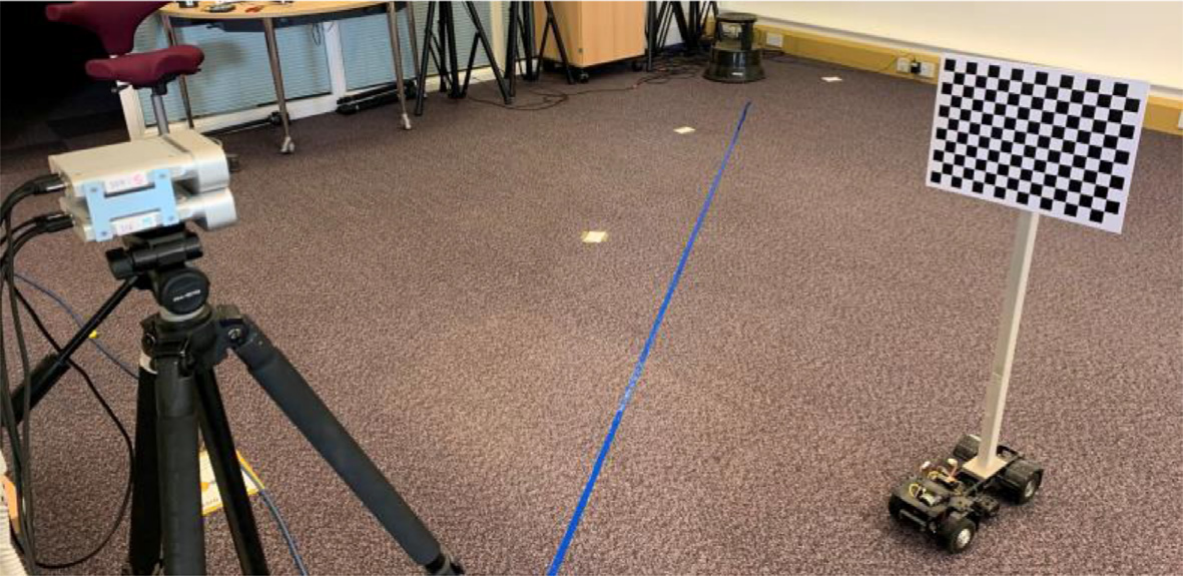
The calibration set-up used in data collection: the A3 board was attached to a movable stand such that its pose could be easily changed.

For the calibration of two Azure Kinect sensors to the same coordinate system, Microsoft provides an open-source Azure Kinect Sensor SDK (found in their GitHub repository [3]) and an example implementation named *green_screen* . The provided SDK seamlessly integrates with OpenCV [6] functions for colour-camera calibration. We based our implementation on the above-mentioned example introducing various extensions. More specifically, the software was modified to use thirty captures instead of one for the calibration, as well as output rotation and translation matrices and RMSE in pixels of the re-projection of the coordinates between Master and Subordinate spaces to a text file. The information can be later used during the post-processing of the original body joint positions of the Subordinate sensor (JSON files) to translate them to the master coordinate space (CSV files). As described in the previous section, the calibration file can be found inside the CALIBRATION directory of the dataset. The file represents a re-projection calculated from the 30 captures. For our calibration, a chessboard-like board was used with predefined dimensions. An A3-sized board was printed on a matte and rigid card to ensure the lowest possible re-projection error. The number of corners used for calibration in the board was 15 *×* 10, and the square size was 25*mm ×* 25*mm*. This board was attached to a movable stand, keeping it completely still while enabling efficient changing of board pose between captures. The set-up is shown in Fig. 5.

**Fig. 6:**
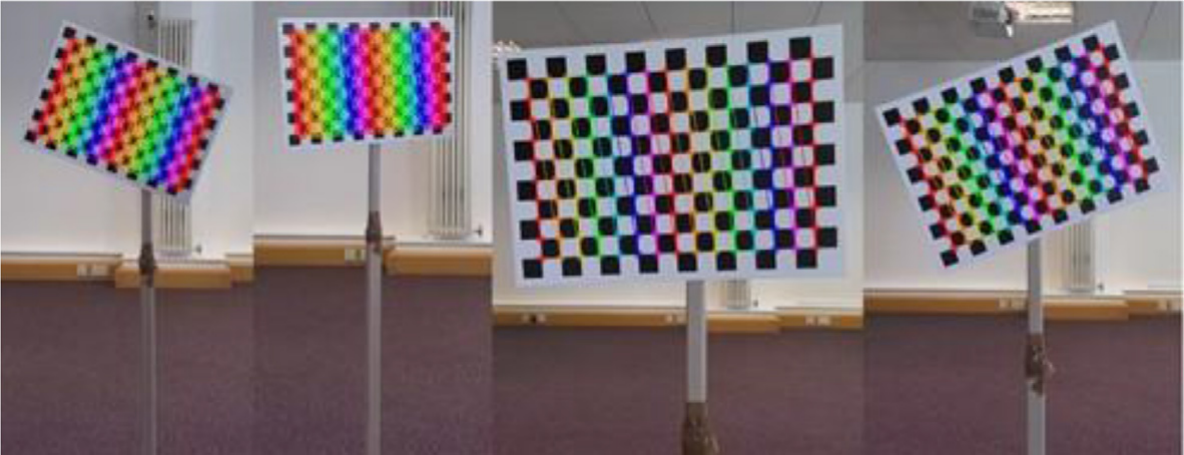
Sample captures taken during calibration, from the perspective of the Master, after drawChessboardCorners() was applied.

Fig. 6 shows example captures taken during the calibration procedure. Using OpenCV's drawChessboardCorners() function [6], we represent the algorithm's prediction of the corner locations. After 30 captures, the re-projection error was stated, and the calibration matrices were placed in a text file. While a lower error is preferable, it must be noted that the RMSE is calculated based on the 2D RGB image plane re-projection, which does not consider the depth axis. The RGB to Depth sensor calibration is factory provided by Microsoft and embedded in the Azure Kinect devices.

**Fig. 7:**
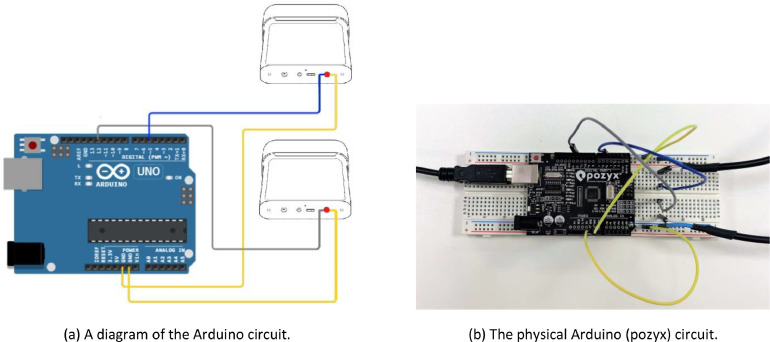
The Arduino circuit used for external synchronisation of the two Azure Kinects. The signal cables (blue and grey) send Sync Out pulses to each device via an auxiliary cord, which is connected to ground via the ground cables (yellow).

**Fig. 8:**
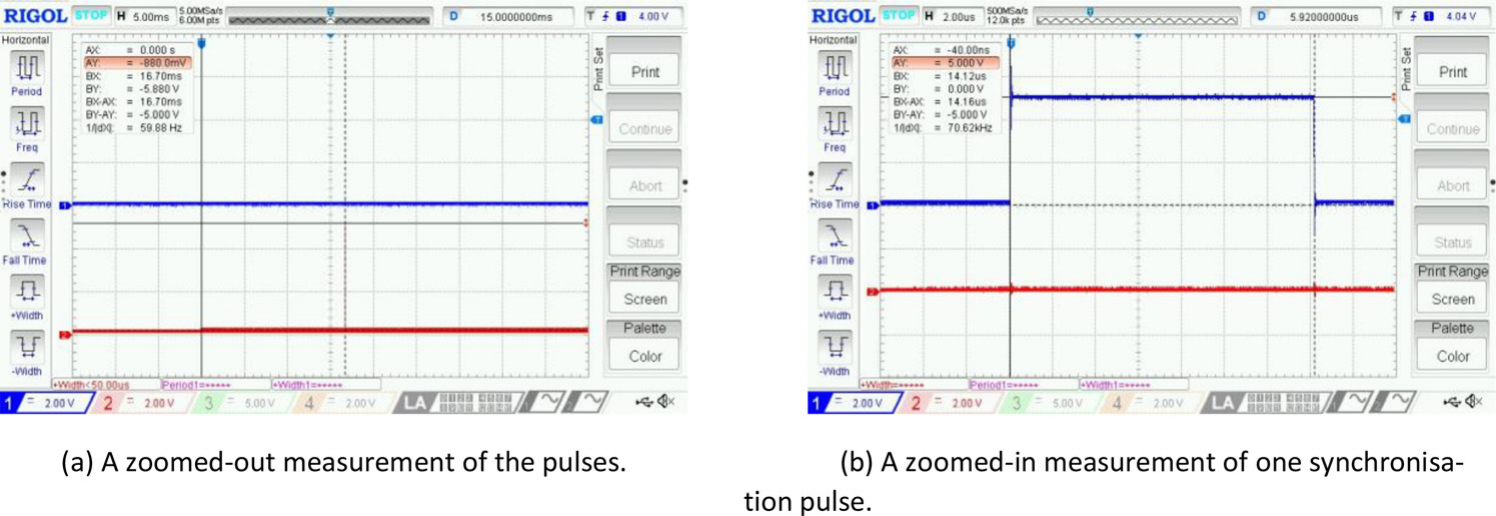
Oscilloscope readings of the Arduino synchronisation pulses

For this dataset, we decided to use the dual-Kinect setup to effectively double the system frame rate. This setup enables the reduction of occlusion during experimentation. To do so, we controlled the timing of each capture on both devices through a synchronisation signal. The signal was sent into their respective Sync In ports and mimicked the Sync Out signal of a Master device. The pulses from the Sync Out port were measured to be around 14*µs*. Microsoft suggests using a signal period of *>* 8*µs*, with 5V active high and 0V passive low [1]; the 5V active high is used as the capture trigger for the depth image by the devices. Based on these specifications, an Arduino Uno^1^ was used to output pulses from two different pins. The Arduino was programmed to emit one pulse per sensor at the start of capture. The circuit used for external synchronisation is shown in Fig. 7. The signals were fed into the Sync In ports of both devices, such that they both acted as Subordinates. This was done using a modified auxiliary cable, in which the internal copper and red wires were heat-shrunk together and grounded, and the white wire was connected to the signal from the Arduino. An oscilloscope was used to verify the frequency and time period of the pulses (Fig. 8). There is some ringing at the start of each pulse, as well as in the other channel, but this was negligible and did not affect the captures.

To confirm the capture timings, we modified the *k*4*arecorder* tool (part of the Azure Kinect Sensor SDK) [3] to output image system timestamps for each captured frame. Comparing the timings, it was validated that the average difference between consecutive device captures was in the correct range, confirming that they were interleaved. Based on that, the doubled frame rate was achieved. The average time difference between consecutive S and M frames for each sequence, according to the system timestamps, is given in “DATASET INFORMATION/frame rate.txt”. The fluctuation of the frame period is likely due to USB bandwidth limitations when the PC receives each image from the device.

**Fig. 9:**
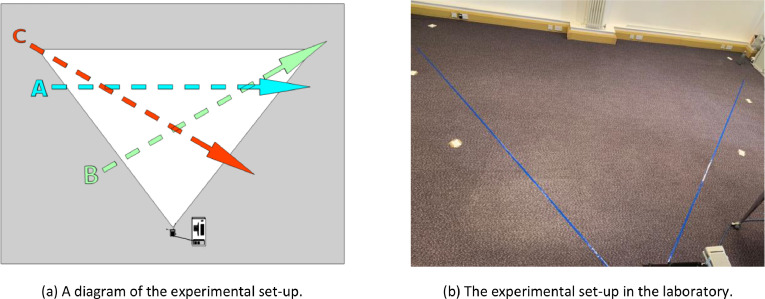
The data collection set-up: the trajectories A, B and C, shown in (a), are represented by floor markers in (b). The triangular walking area represents the Kinect's FoV in NFOV Unbinned mode.

When capturing the dataset, participants walked either orthogonally to the sensors at roughly 3*m* distance, or at *±*30° to the normal line-of-sight, between edges of the FoV. The trajectories can be seen in Fig. 9. The actions performed and their corresponding code names are as follows:

• Regular walking style (normal).
• Regular walking style in the opposite direction (opposite).
• Taking a phone call (call).
• Texting on a phone (text).
• Holding a drink in one hand (drink).
• Carrying a briefcase (carry).
• Wheeling a suitcase (case).

**Fig. 10:**
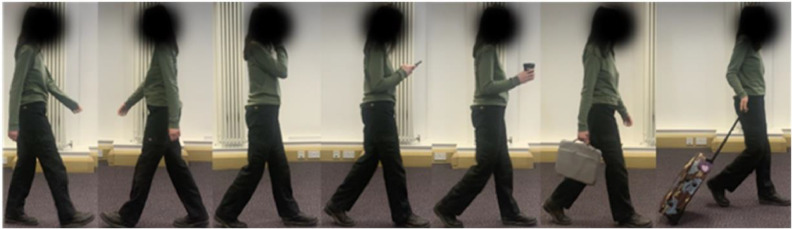
The action classes used in data collection, ordered from left-to-right: normal, opposite, call, text, drink, carry, case.

Example frames of each action are shown in Fig. 10. Each action was performed three times (once per trajectory) and captured from both sensors. This resulted in 21 walking sequences per participant and two videos per sequence. Participants were assigned numbers to ensure their anonymity. The recordings were captured using the modified version of *k*4*arecorder*. The dataset consists of 15 participants and, therefore, 630 30fps sequences, or 315 interleaved sequences at 60fps.

**Fig. 11:**
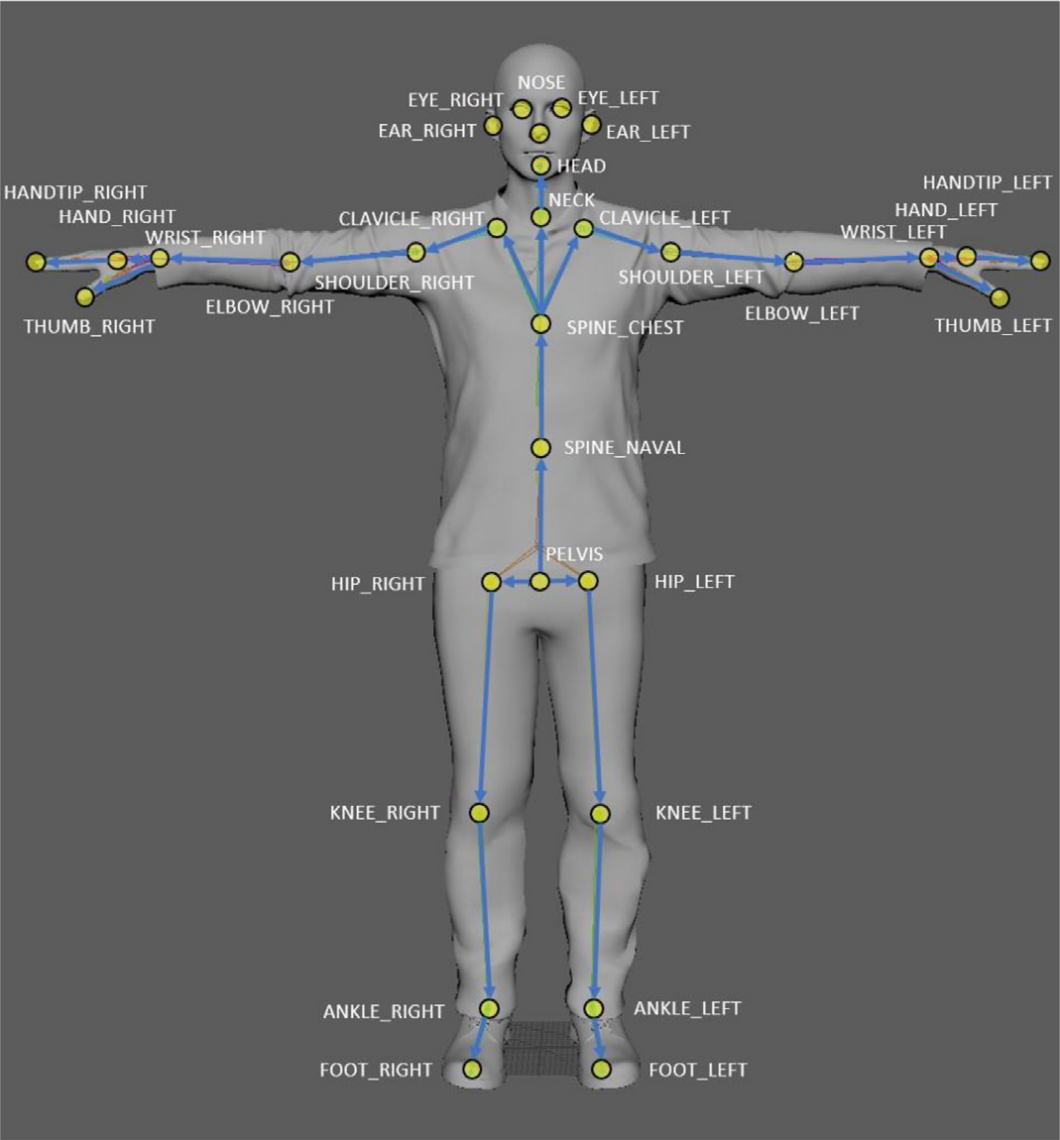
A diagram of the 32 joints tracked by the Body Tracking SDK, from the Azure Kinect documentation [1].

The final step of our processing is based on obtaining the skeletal information for each frame. Microsoft's *offline_processor* [4] tool was modified to output coordinates according to the colour coordinate system rather than depth, such that the RGB calibration file can be applied. The program outputs a JSON file containing body tracking data. After feeding the 630 video sequences to the tool, we obtain the 630 JSON files on this dataset that provide the joint coordinates recognized by the Azure Kinect (Fig. 11) referenced to each camera's RGB coordinate spaces. These, alongside the 630 image system timestamp TXT files for the different sensor outputs (RDG, Depth, IR), are found in the SUBJECTS directory of the dataset.

The 630 JSON files must be referenced in a single coordinate space per capture. This involves translating one of the device's coordinate spaces to the second device's space. As mentioned previously, in our case, we decided to reference the Subordinate sensor to the Master device's RGB coordinate space via the calibration file. This outputs the 630 CSV files that provide the body joints detected by the two Azure Kinect devices over the 315 sequences in the same coordinate space. Combined with the TXT files, we have these joint detections referenced to a common system time.

## Ethics Statements

The work involved human subjects who signed a release document authorizing the use of their image by Toshiba Research Europe for purposes like publication of processed data like the one described in this document. Informed consent was provided by the participants of the experiment. Ethical approval from an ethics committee was not required nor obtained.

## CRediT authorship contribution statement

**Charli Posner:** Conceptualization, Investigation, Software, Data curation, Writing – original draft. **Adrián Sánchez-Mompó:** Conceptualization, Software, Supervision, Writing – review & editing. **Ioannis Mavromatis:** Writing – review & editing. **Mustafa Al-Ani:** Conceptualization, Supervision.

## Declaration of Competing Interest

The authors declare that they have no known competing financial interests or personal relationships that could have appeared to influence the work reported in this paper.

## Data Availability

Azure Kinect Body Tracking Dataset (Original data) (Zenodo)

## Footnotes

1 We used a Pozyx microcontroller board, which is architecturally similar to the Arduino Uno
